# Oral Manifestations Related To CD4 Lymphocyte Count in HIV-Positive Patients

**DOI:** 10.5681/joddd.2010.029

**Published:** 2010-12-21

**Authors:** Poorandokht Davoodi, Mina Hamian, Reza Nourbaksh, Fatemeh Ahmadi Motamayel

**Affiliations:** ^1^ Assistant Professor, Department of Oral Medicine, Dental School, Hamadan University of Medical Sciences, Hamadan, Iran; ^2^ Post-graduate student, Department of Oral Medicine, Dental School, Shahid Beheshti University of Medical Sciences, Tehran, Iran; ^3^ Dentist, Hamadan, Iran; ^4^ Assistant Professor, Department of Oral Medicine, Dental School, and Research Center for Molecular Medicine, Hamadan University of Medical Sciences, Hamadan, Iran

**Keywords:** CD4, HIV positive, oral manifestation

## Abstract

**Background and aims:**

The onset of opportunistic infections in HIV-positive patients is generally associated with a low CD4 count. Oral manifestations can be the first clinical sign of the infection and also determine the progression of disease. The purpose of this study was to determine the prevalence of oral soft tissue manifestations and their relationship with the degree of immunosuppression observed in HIV-positive patients.

**Materials and methods:**

100 HIV-positive patients were examined. Oral lesions were evaluated according to EEC clearing house criteria. The degree of immunosuppression was based on the CD4 count closest to the oral examination. Data were analyzed using Student’s t-test and chi-square test.

**Results:**

The most common oral lesions were rampant caries (54%), periodontal disease (44%), and hyperpigmentation (42%). Salivary glands enlargements and leukoplakia were associated with more severe immunosuppression.

**Conclusion:**

According to the results, it seems that occurrence of only some of oral lesions are related to the degree of immunosuppression and such lesions can be considered as indicators of the progression of the HIV infection.

## Introduction

Acquired immunodeficiency syndrome (AIDS) epidemic is increasing all over the world without any definite treatment. Antiretroviral medications can only control the progress rate of the disease.^1-3^ Oral lesions might be considered as the initial manifestations of the disease. Oral manifestations of HIV infection are important in the AIDS epidemic and some of them could be used to assess the status of immunosuppression and determine the prognosis of the disease.-^7^ Some oral lesions may even alter patients’ quality of life.^8^ Early diagnosis and appropriate treatment of oral lesions have great influence on patients’ general health and can reduce the mortality rate of the disease.^9^ Although some lesions such as candidiasis and hairy leukoplakia are considered as prognostic factor, recent data have indicated that concurrent existence of multiple and variable oral lesions is accompanied with poor prognosis of the disease.^10^ Reduction of circulating CD4 count is the main criteria for assessing the immunosuppression status in HIV-positive patients. The number of circulating CD4 cells ranges from 600 to 1600 cells/mm^3^, but the initial signs of immunosuppression occur when CD4 count is lower than 500 cells/mm^3^.

Studying the prevalence of AIDS-related oral lesions in different regions of the world is important as it provides a more thorough description of the epidemic and scientific basis for the appropriate management of the disease by health care workers.^8^ Santos et al^3^ reported that oral lesions were common among HIV+ children and the most common lesion was pseudomembranous candidiasis. Most of the patients with oral candidiasis were suffering from severe immunosuppression, and they concluded that candidiasis and gingivitis could be considered as an indicator of the disease progress.^3^ Another study on HIV-related oral lesions and complications in two hospitals Iran showed that the most common oral manifestations were erythematous candidiasis and angular cheilitis.^4^ An evaluation of 237 HIV-positive patients revealed high prevalence of oral lesions, with candidiasis, oral hairy leukoplakia, and HIV-related periodontal disease being three most common manifestations, and candidiasis significantly associated with low CD4 count (less than 200).^9^ Other studies have also shown an association between low CD4 counts and candidiasis, linear gingival erythema, angular cheilitis, scabies, paronychia, oral pigmentation, diffuse hair loss and other oral symptoms.^11,12^

Since the oral manifestations of AIDS are common and can be considered as indicators of immunosuppression, the aim of this survey was to investigate if there was a relationship between oral manifestations and the level of immunosuppression in HIV-positive patients. The results might be used as a guide in determining the immunosuppression level of AIDS according to oral manifestations.

## Materials and Methods

A cross-sectional study was carried out among 100 proven HIV-positive patients, selected randomly from those attending Behavior Consultation Center of Kermanshah, Kermanshah Province, Iran. Patients were informed about the objective of the study and signed an informed consent form before oral examination. There was no control group in this study.

Data including sexual habits, IV drug abuse, hemophilia, blood transfusion, addiction, other diseases, previous CD4 counts, quantification of viral load and opportunistic infections were extracted from the patient files. All patients received oral hygiene instructions. CD4 counts were determined using complete blood count and flow cytometry.

A questionnaire was completed for each patient regarding past medical history, systemic signs and symptoms, and oral manifestations. Patients were examined by an infectious disease specialist. Oral cavity was inspected carefully. Intra-oral examination was performed by a dentistry student under supervision of an oral medicine specialist on a dental unit using disposable dental mirrors and sterile gauze pads under appropriate lighting. Submandibular, submental and cervical lymph nodes were palpated bidigitally by the examiner. Further clinical examination using an explorer and probing with a periodontal probe were done in order to diagnose dental caries and periodontal diseases. The supervision of the oral medicine specialist continued during the examinations. All patients were examined by similar instruments, under the same circumstances and at the same place.

The diagnosis was based upon ECC clearing house’s criteria, and diagnostic criteria for common HIV-related oral lesions such as pseudomembranous and erythematous candidiasis, angular cheilitis, linear gingival erythema, Kaposi sarcoma, hairy leukoplakia and recurrent aphthous stomatitis. The level of immunosuppression in each patient was classified according to CDC classification by CD4 count and based on WHO immunologic staging as absent (>800/ ml), moderate (>500/ml) and severe (<200/ml).^12- 15^

The SPSS 10.0 computer software was used for data analysis. To compare CD4 counts in individuals with and without specific lesions, t-test was employed. In order to compare the proportions of immunosuppression, chi-square test was applied to data. P < 0.05 was considered statistically significant.

## Results

100 patients (mean age, 34±7.7 years old; 92% males) were investigated. The mean duration of infection was 5.4±3.2 years. History of imprisonment (85%), body tattoo (81%), drug abuse (92%) and IV drug abuse (85%), blood transfusion (2%), traveling abroad (5%), and high risk sexual behaviors (54%) were noted among the studied subjects. Medical history indicated taking anti-tuberculosis medication (PTT positive, 25%) and constant use of antibiotics (40%), treatment for pneumocystis carini (9%) and anti-fungal therapy (4%) and highly active antiretroviral therapy (HAART, 15%).

The most common observed oral manifestations were rampant caries, periodontal diseases, hyperpigmentation, and erythematous candidiasis (Table 1).

**Table 1:** Prevalence of oral lesions in the evaluated HIV-positive patients (n = 100)

Lesion	Number	Percent
Rampant caries	54	54%
Periodontal disease	44	44%
Hyperpigmentation	42	42%
Erythematous candidiasis	36	36%
Xerosthomia	20	20%
Angular cheilitis	17	17%
Leukoplakia	16	16%
Hairy tongue	14	14%
Salivary gland enlargement	11	11%
Pseudomembranous candidiasis	7	7%
Linear gingival erythema	6	6%

The mean count of CD4 + lymphocytes was 542±34 (range, 66–1663) and 43 patients were not severely immunosuppressed. The frequency of HIV-related oral manifestations according to severity of immunosuppression in the studied population is shown in Table 2. Salivary gland enlargement, hairy tongue and leukoplakia were accompanied with more severe immunosuppression.

**Table 2:** Frequency (percent) of HIV-related oral manifestations according to severity of immunosuppression in the studied population (n = 100)

Lesion	Number	Severity of immunosuppression in patients with this type of lesion			Severity of immunosuppression in patients without this lesion			Chi2	P value
		No suppression	Moderate	Severe	No suppression	Moderate	Severe		
Rampant caries	54	23 (42.6%)	25 (46.3%)	6 (11.4%)	21 (45.7%)	18 (39.1%)	7 (15.2%)	0.2	0.715
Periodontal disease	44	17 (38.6%)	23 (53.3%)	4 (9.1%)	27 (48.2%)	20 (35.7%)	9 (16.1%)	3.08	0.222
Hyperpigmentation	42	21 (50%)	14 (33.3%)	7 (16.7%)	23 (39.7%)	29 (50%)	6 (10.3%)	2.91	0.233
Erythematous candidiasis	36	15 (41.7%)	14 (38.9%)	7 (19.4%)	29 (45.3%)	29 (45.3%)	6 (9.4%)	20.8	0.352
Xerosthomia	20	10 (50%)	6 (30%)	4 (20%)	34 (42.5%)	37 (46.3%)	9 (11.3%)	2.1	0.345
Angular cheilitis	17	8 (47.1%)	5 (29.4%)	4 (23.5%)	36 (43.4%)	38 (45.7%)	9 (10.8%)	2.67	0.263
Leukoplakia	16	5 (31.3%)	7 (43.8%)	4 (25%)	39 (46.4%)	36 (42.9%)	9 (10.7%)	2.81	o.285
Hairy tongue	14	6 (42.9%)	8 (57.1%)	0	38 (44.2%)	35 (40.7%)	13 (15.1%)	2.87	0.237
Salivary glands enlargement	11	1 (9.1%)	7 (63.7%)	3 (27.3%)	43 (48.3%)	36 (40.4%)	10 (11.2%)	6.58	0.037
Pseudomembranous candidiasis	7	5 (71.4%)	0	2 (28.6%)	39 (41.9%)	43 (46.2%)	11 (11.8%)	5.92	0.052
Linear gingival erythema	6	4 (66.7%)	1 (16.7%)	1 (16.7%)	40 (42.6%)	42 (44.7%)	12 (12.8%)	1.81	0.398

The frequency of oral manifestations and CD4 count in HIV-positive patients are shown in Table 3. Other observed manifestations (less than 5 cases) included hairy leukoplakia (4 cases), lichen planus of tongue (4 cases), fissured tongues (3 cases), necrotizing ulcerative periodontitis (2 cases), oral warts (2 cases), irritation fibromas (2 cases) and papilloma (1 case).

**Table 3:** The comparison of CD4 counts in patients with and without specific oral lesions

lesion	Frequency of patients	Average CD4 count patients with this lesion	Average CD4 count in patients without this lesion	T	P
Rampant caries	54	54±323	366±545	0.082	0.983
Periodontal diseases	44	297±523	375±557	0.490	0.625
Hyperpigmentation	42	252±561	337±529	0.462	0.664
Erythematous candidiasis	36	284±461	365±584	1.805	0.074
Xerostomia	20	336±518	345±584	0.349	0.728
Angular cheilitis	17	310±460	384±559	1.089	0.279
Leukoplakia	16	407±496	330±551	0.554	0.560
Hairy tongue	14	517±760	294±507	2.643	0.010
Salivary gland enlargement	11	156±315	349±570	-2.385	0.019
Pseudomembranous candidiasis	7	484±663	331±533	-0.96	0.336
Linear gingival erythema	6	299±652	346±541	0.145	0.885

## Discussion

Prevalence of AIDS-related oral manifestations is different among studies. The main reasons of this difference are variable sample size, the stage of the disease, the degree of immunosuppression, inter-examiner differences, and regional patterns of infectious diseases. Therefore, extrapolating the results comparing the reported prevalence of existing studies may not very informative and careful diagnosis and treatment of oral lesions is recommended.

In the present study, the most common oral manifestations of AIDS were rampant caries, periodontal disease, hyperpigmentation and erythematous candidiasis. More than 50% of patients had rampant caries while only 36% showed erythematous candidiasis. Previous studies, however, reported pseudomembranous candidiasis as the most common oral manifestation.^3,4^ The prevalence of pseudomembranous candidiasis varies from 38 %,^5^ to 17%,^4^ to 7% in the present study. This difference can be due to differences in the prescribed medications, the stages of the disease or the way of transmission of the infection, and needs more investigations. Although immunosuppression is commonly accompanied by fungal infections,,^16, 17^ there was no significant relationship between CD4 count in patients with candidiasis (both pseudomembranous and erythematous ) and other patients in the present study. Some possible reasons are co-existence of other lesions or the effect of HAART, which can control candidiasis with an indirect effect on CD4+ count. Recent studies have indicated a definite relationship between pseudomembranous candidiasis (mostly erythematous) and progressing HIV infection.^3,4,11^ In our study, CD4+ count was almost the same in patients with angular cheilitis (460±310) and erythematous candidiasis (461±284), both lower than that in other patients. However, immunosuppression was more severe in patients with pseudomembranous candidiasis (663±484). The prevalence of candidiasis in this study was less than that in other studies, but the severity of immunosuppression in patients with candidiasis was higher.^18^ In the present study, rampant caries was the most common bacterial infection with a mean CD4 count of 323±54 among affected individuals, which indicates a milder immunosuppression degree. Rampant caries and severe periodontal diseases (mean CD4 count, 523±297) might have caused tooth loss and dentures use in some patients with severe immunosuppression, resulting in not being categorized as rampant caries.

Bruce showed that a decreasing CD4 lymphocyte count was associated with an increasing severity of gingival disease.^15^ Periodontal disease was another oral manifestation in patients with lower CD4 count in the present study. It may be considered as a result of poor oral hygiene, drugs, medications and alcoholic beverages. Hyperpigmentation is a non-characteristic manifestation of AIDS that is caused by HAART, smoking and addiction as well as adrenal insufficiency the latter, however, was not seen in our study.^19^

Patients with salivary gland enlargement suffered from severe immunosuppression (315±156) and their CD4+ count was lower than other patients. Linear gingival erythema (652±299) is not characteristic of AIDS and lack of a significant relation between this lesion and CD4 count was justified. The results indicated that salivary gland enlargement (315±156), dental caries (323±54), angular cheiliti (460±310), and erythematous candidiasis were accompanied by the least CD4 count, respectively. CD4 count was in an intermediate level in the case of other lesions.

The highest level of immunosuppression was noticed in the following groups, respectively: pseudomembranous candidiasis (28.6%), salivary gland enlargement (27.3%), leukoplakia (25%), and angular cheilitis (23.5%). Coated tongue (760±517) was the most prevalent condition in HIV-positive patients with intermediate CD4 count. According to the fact that this condition can also occur in otherwise healthy people, this result could be justified.^20^

Despite the relationship between oral manifestations and CD4 count, other aspects such as the stage of the disease, the way of the transmission of infection, and the success rate of the prescribed medications are different in various societies. Additional studies are needed in other parts of the world to determine the epidemiology of oral lesions and their relation with immunosuppression and the way of the transmission of infection.

Because of the limited number of accessible HIV-positive patients, even those under treatment were not excluded from the study. In future studies, other variables such as the way of transmission of the infection, the stages of the disease, and HAART should be considered in the study design.

## Conclusion

According to the results, the most common observed oral lesions were, in descending order, rampant caries (54%), periodontal diseases (44%), hyperpigmentation (42%), and erythematous candidiasis (36%). Salivary gland enlargement, rampant caries, and leukoplakia revealed significant relationship with the degree of immunosuppression and CD4 count compared to other oral manifestations of AIDS.
